# Antimicrobial activity of resveratrol-derived monomers and dimers against foodborne pathogens

**DOI:** 10.1038/s41598-019-55975-1

**Published:** 2019-12-20

**Authors:** Luce M. Mattio, Sabrina Dallavalle, Loana Musso, Rossella Filardi, Laura Franzetti, Luisa Pellegrino, Paolo D’Incecco, Diego Mora, Andrea Pinto, Stefania Arioli

**Affiliations:** Department of Food, Environmental and Nutritional Sciences (DeFENS), University of Milan, Via Celoria 2, 20133 Milan, Italy

**Keywords:** Medicinal chemistry, Antimicrobials, Antimicrobial resistance

## Abstract

Plant polyphenolic compounds are considered a promising source for new antibacterial agents. In this study, we evaluated the antimicrobial activity of a collection of resveratrol-derived monomers and dimers screened as single molecules against a panel of nine foodborne pathogens. The results demonstrated that two monomers (i.e., pterostilbene **2** and (*E*)-3-hydroxy-4′,5-dimethoxystilbene **9**) and three dimers (i.e., *δ*-viniferin **10**, viniferifuran **14** and dehydro-*δ*-viniferin **15**) were endowed with significant antibacterial activity against gram-positive bacteria. The exposure of gram-positive foodborne pathogens to 100 µg/mL of **2**, **9** and **15** induced severe cell membrane damage, resulting in the disruption of the phospholipid bilayer. The most promising dimeric compound, dehydro-*δ*-viniferin **15**, was tested against *Listeria monocytogenes*, resulting in a loss of cultivability, viability and cell membrane potential. TEM analysis revealed grave morphological modifications on the cell membrane and leakage of intracellular content, confirming that the cell membrane was the principal biological target of the tested derivative.

## Introduction

**Figure 1:**
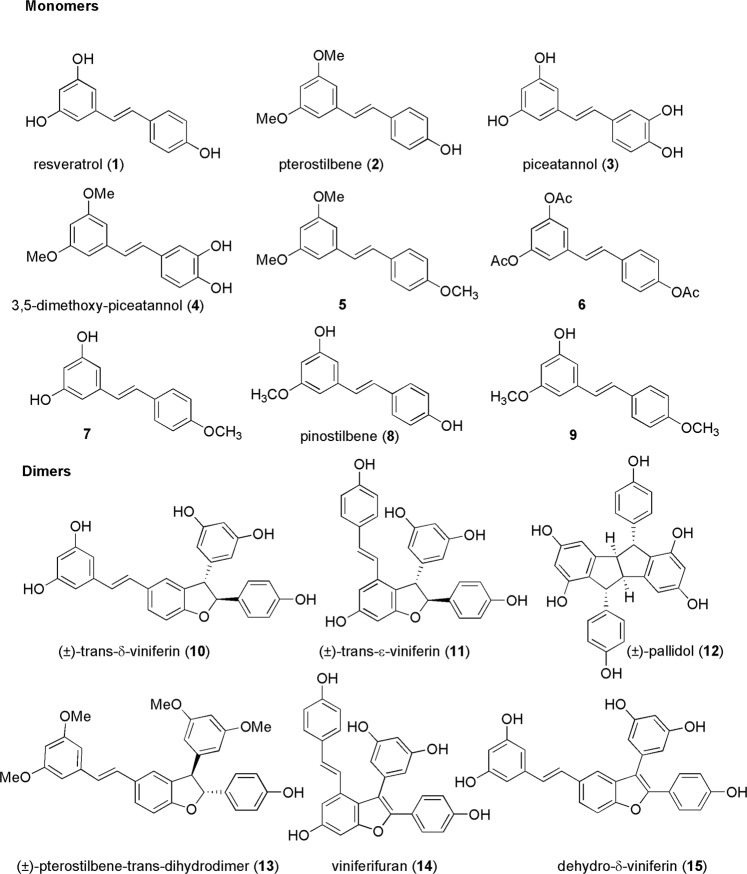
Structures of stilbenoid monomers (**1–9**) and dimers (**10–15**).

Food contamination with pathogens is a common health concern. Foodborne illnesses are the consequence of the ingestion of foods or drinks that are contaminated with pathogenic microorganisms, such as *Listeria monocytogenes*, *Salmonella enterica*, *Staphylococcus aureus*, and *Bacillus cereus*, which are among the leading causative agents^1,2^. Due to the intensive use or overuse of antimicrobial agents and interrupted treatments in humans and animals, bacteria may develop resistance towards antibiotics, which currently represents a recognized public health problem worldwide^3,4^. Indeed, infections due to multidrug resistant pathogens could lead to a great risk of morbidity and mortality. Bacteria can develop resistance by horizontal gene transfer, specific gene mutations or several mechanisms such as efflux pump activity, cell wall impermeability, and synthesis of antibiotic degrading enzymes^5,6^. In this scenario, natural product research is ready to regain prominence in the supply of novel molecules to solve the antibiotic crisis^7–9^. Natural products have been the main source of antimicrobials for centuries, and plant extracts have been successfully used for treating several illnesses^10–12^. The chemical diversity of natural compounds that still have to be effectively explored is tremendously large, and identifying novel antimicrobials and understanding their mechanism of action are equally imperative tasks. Resveratrol and resveratrol-derived monomers and oligomers (stilbenoids), isolated from grapes, peanuts, cranberries, and other botanical sources^2,13,14^, are endowed with multifaceted biological activities^15^. The interest in the pharmacological potential of resveratrol-derived compounds has recently increased due to both the poor bioavailability and the incomplete understanding of the pharmacodynamics of the parent molecule, which severely hampers its therapeutic applications^16^. Stilbenoids, like many other secondary metabolites, are chiefly expressed as biological defense compounds in response to pathogenesis^15,17,18^. For this reason, the investigation of their antimicrobial activity is of great interest. In particular, stilbenoids have shown significant antibacterial activity against gram-positive rather than gram-negative bacteria^2,19^. However, systematic studies are quite scarce and incomplete since investigators often assay natural extracts as complex mixtures of compounds and not as pure molecules. Indeed, isolation from plant material usually yields only minimal quantities of a single compound through laborious extraction and extensive purification processes. Therefore, access to these natural products by chemical synthesis is instrumental to overcome the limitations imposed by material scarcity. The research described herein details our efforts to investigate in detail the antimicrobial activity and mode of action of a focused collection of simple stilbenoids (**1**–**15**, Fig. 1). The compounds were selected on the basis of differences in the substitution pattern (-OH, -OCH_3_, -OCOCH_3_) of the aromatic rings and the shape and geometry of the molecules. Suitable amounts of these molecules for rigorous biological testing were made available by combining reported chemoenzymatic synthetic strategies^20–28^. This approach allowed us to provide an integrated overview on the antimicrobial activity of this class of compounds against foodborne pathogenic species.

## Results

### Synthesis of polyphenolic compounds

**Figure 2:**
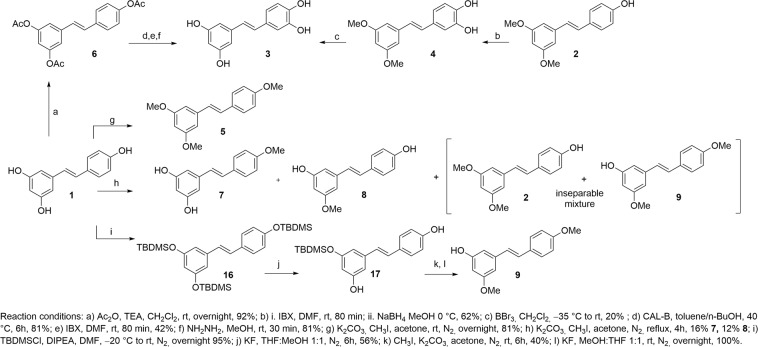
Synthesis of resveratrol-derived monomers.

To provide new insight into the antimicrobial activity of resveratrol derivatives, we planned to perform preliminary structure-activity relationship (SAR) studies on this class of compounds. Among the representative stilbene monomers (compounds **1**–**9**, Fig. 2), both resveratrol **1** and pterostilbene **2** were commercially available at a low cost. Attempts to prepare piceatannol **3** by a regioselective aromatic oxidation of resveratrol **1** with iodoxybenzoic acid (IBX) in MeOH at −78 °C, followed by the reduction of the intermediate *o*-quinone by Na_2_S_2_O_4_ resulted in inseparable complex product mixtures^20^. Attempts to obtain piceatannol starting from pterostilbene **2** through the formation of the oxidized intermediate **4** gave the desired product **3** in modest overall yield (12%) (Fig. 2).

Alternatively, the chemoenzymatic approach described by Bernini *et al*.^21^ allowed to obtain piceatannol **3** starting from compound **6**, on its turn obtained by peracetylation of resveratrol **1** (25% overall yield) (Fig. 2).

Monomers **5**–**9** were all obtained by methylation of resveratrol **1** (Fig. 2). Compound **5** was prepared using K_2_CO_3_ and CH_3_I (9 eq) under a N_2_ atmosphere at room temperature overnight in the dark, whereas derivatives **7** and **8** were obtained by the partial methylation of resveratrol **1** using K_2_CO_3_ (2 eq.) and CH_3_I (2 eq.) for 4 h under reflux^22^. This reaction also led to the formation of product **9** (3,4′-dimethoxyresveratrol), which was recovered together with its inseparable regioisomer **2** (pterostilbene). Therefore, a different synthetic strategy was developed to obtain **9** as a pure compound. Resveratrol **1** was converted into tri(*t-*butyldimethylsilyl)ether **16** (95%), which was selectively deprotected at positions 3 and 4′ by KF (1 eq) at −15 °C for 6 h (56%)^23^. The obtained compound **17** was subsequently methylated and deprotected to afford **9** as a single isomer (40% over 2 steps).

Having monomers **1**–**9** in our hands, we next focused on dimeric compounds. Resveratrol dimers are almost universally generated by oxidative radical coupling^24^. Thus, we followed a biomimetic synthetic strategy that allowed us to obtain dimers **10**–**15** (Fig. 1) in sufficient quantities (scalable to the order of grams) for their systematic biological evaluation as antimicrobials.

**Figure 3:**
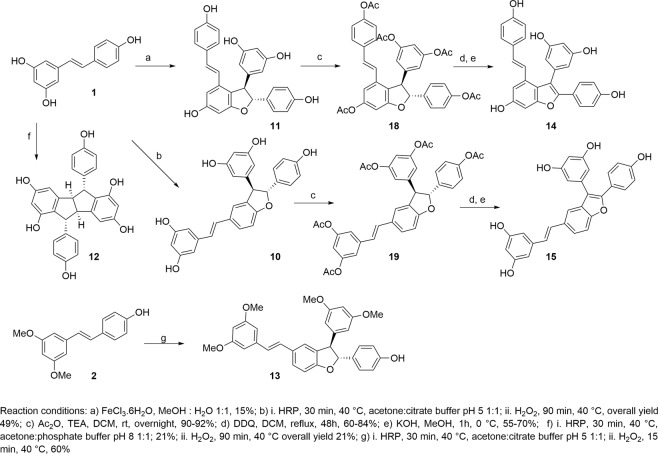
Synthesis of resveratrol-derived dimers.

Using the method developed by Li *et al*.^25^, resveratrol **1** was treated with HRP (1 mg/mL solution) and H_2_O_2_ (30% solution) in a solvent system based on a 1:1 (v/v) mixture of acetone and aqueous buffer (Fig. 3). Enzymatic activity is greatly influenced by the environmental pH^26^. Indeed, under acidic conditions (citrate buffer, pH 5), trans-*δ*-viniferin **10** was selectively formed in 49% yield, whereas under basic conditions (phosphate buffer, pH 8), pallidol **12** was the main product (21% yield), recovered with minor amounts of *trans*-*δ*-viniferin (10%). On the other hand, *trans*-*ε*-viniferin **11** was obtained through metal-catalyzed oxidative coupling. By treating resveratrol **1** with the electron oxidant Fe^3+^(FeCl_3_.6H_2_O) in a MeOH:H_2_O solvent system, the desired product **11** was obtained in 15% yield^27^.

Starting from *trans*-*ε*-viniferin (**11**) and *trans*-*δ*-viniferin (**10**), the corresponding benzofuran derivatives **14** and **15** were obtained. Acetylation using Ac_2_O and TEA in DCM gave peracetylated compounds **18** and **19**, which were subjected to oxidative dehydrogenation with DDQ (20 eq) in DCM at reflux for 48 h^28^. Final deprotection of the phenolic moieties with KOH in MeOH at 0 °C gave the natural product viniferifuran (**14**) and its regioisomer *trans*-dehydro-*δ*-viniferin (**15**) in 55% and 70% overall yield, respectively (Fig. 3). Finally, pterostilbene-*trans*-dihydrodimer (**13**) was obtained in 60% yield by treatment of pterostilbene **2** with horseradish peroxidase (HRP/H_2_O_2_) at pH 5 (citrate buffer), (Fig. 3).

### Antimicrobial activity of polyphenolic compounds against *Staphylococcus aureus* DSM 25923 and *Pseudomonas aeruginosa* ATCC 27853

The synthesized molecules **1**–**15** were tested against foodborne pathogens, namely, *S. aureus* ATCC 25923 and *P. aeruginosa* ATCC 27853, as representatives of gram-positive and gram-negative bacteria, respectively. The minimum inhibitory concentration (MIC) and minimum bactericidal concentration (MBC) values showed that, out of the 9 monomeric compounds (**1** to **9**), only pterostilbene **2** and its regioisomer **9** had antimicrobial activity against *S. aureus* at relatively low concentrations, with MICs of 4 μg/mL and 64 μg/mL, respectively (Table 1). Interestingly, the MIC of pterostilbene **2** was approximately 128-fold lower than that of resveratrol **1** (512 µg/ml). When tested against *P. aeruginosa*, monomeric compounds **3**, **7**, **8** and **9** showed MIC values of 64–128 μg/mL, suggesting a reduced susceptibility of gram-negative microorganisms towards these compounds. Nevertheless, MBC values (≥512 μg/mL) for most of the tested monomers indicated a growth inhibitory rather than bactericidal effect on both gram-positive and gram-negative bacteria. Dimers **10–15** were also tested. Notably, dimers **10**, **12**, **14**, and **15** were endowed with a relevant activity on gram-positive *S. aureus*, with MIC values of 16, 64, 16 and 2 μg/mL, respectively, which were much lower than that of the parent compound resveratrol **1** (512 µg/mL). Conversely, the sensitivity of gram-negative *P. aeruginosa* to the dimers tested on was modest (128–256 μg/mL). As observed for the monomers, the MBC values (≥512 μg/mL) of dimers **10** and **14** on both *S. aureus* and *P. aeruginosa* confirmed a prevailing inhibitory effect. Interestingly, compound **15** was able to induce *S. aureus* cells death at low concentrations (16 µg/mL).

**Table 1:** Antimicrobial activity of compounds 1–15 against *S. aureus* and *P. aeruginosa*.

Cpd	*S. aureus* ATCC 25923		*P. aeruginosa* ATCC 27853	
	MIC (µg/mL)	MBC (µg/mL)	MIC (µg/mL)	MBC (µg/mL)
1	512	>512	>512	>512
2	4	128	512	512
3	>512	>512	128	>512
4	128	512	512	>512
5	>512	>512	>512	>512
6	>512	>512	>512	>512
7	256	>512	64	>512
8	512	>512	64	>512
9	64	512	128	256
10	16	512	256	>512
11	512	>512	256	>512
12	64	>512	>512	>512
13	>512	>512	>512	>512
14	16	>512	128	>512
15	2	16	128	>512

### Assessment of susceptibility of foodborne pathogens against resveratrol derivatives

Based on the MIC and MBC values (Table 1), we selected monomers **2** and **9** and dimers **10**, **14** and **15** for further investigation. As a reference molecule, we included chlorhexidine, an active biocide against both gram-positive and gram-negative bacteria, in this screening (Table 2)^29^. The antimicrobial activity of the selected compounds was evaluated against nine species of foodborne pathogens, five belonging to the gram-positive group (*L. monocytogenes* ScottA, *S. aureus* ATCC 25923, *E. faecium* DSM 20477, *E. faecalis* DSM 20478, and *B cereus* DSM 9378), and four belonging to the gram-negative group (*P. aeruginosa* ATCC 27853, *E. coli* DSM 682, *E. coli* DSM 8579, *S. enterica* DSM 9386, and *Proteus hauseri* DSM 30118). The MIC and MBC values clearly confirmed a higher susceptibility of gram-positive bacteria towards the selected dimers **10**, **14** and **15** (Table 2). Indeed, for these molecules, the MIC values were generally lower than those observed for pterostilbene **2** (4–64 µg/mL). In particular, the MIC values were 4–16 µg/mL for both **10** and **14** and reached 1–4 µg/mL for **15**. Compound **9** was less effective and more species-dependent than its regioisomer pterostilbene **2**, with MIC values assessed at 4–256 µg/mL. The MBC of **10** and **14** (128–512 µg/mL) was comparable to that of pterostilbene **2** (128–512 µg/mL), whereas interestingly, these MBC values were higher than that of compound **15** (16–64 µg/mL). When tested against gram-negative bacteria, the selected compounds showed MIC and MBC values of 128–512 µg/mL and ≥512 µg/mL, respectively, confirming a reduced susceptibility of this group of bacteria towards the studied stilbenoids^30^. Conversely, chlorhexidine was efficiently active at low concentrations against both gram-positive and gram-negative bacteria (MIC 4–32 µg/mL, MBC 16–128 µg/mL) (Table 2).

**Table 2:** MIC and MBC (in brackets) values of selected monomers **2**, **9** and dimers **10**, **14**, **15** against Gram-positive and Gram-negative bacteria.

	MIC (MBC) µg/mL					
	14	15	10	9	2	Chlorhexidine
**Gram-positive**						
*L. monocytogenes* Scott A	16 (>512)	2 (16)	16 (128)	256 (512)	64 (128)	8 (32)
*S. aureus* ATCC 25923	16 (>512)	2 (16)	16 (512)	64 (512)	4 (128)	32 (128)
*E. faecium* DSM 20477	8 (>512)	2 (32)	8 (512)	4 (512)	32 (512)	4 (128)
*E. faecalis* DSM 20478	8 (512)	4 (64)	16 (512)	128 (512)	32 (128)	8 (128)
*B. cereus* DSM 9378	4 (128)	1 (16)	4 (256)	8 (>512)	16 (512)	8 (16)
**Gram-negative**						
*P. aeruginosa* ATCC 27853	128 (>512)	256 (512)	256 (>512)	128 (256)	512 (512)	32 (64)
*E. coli* DSM 682	256 (>512)	512 (>512)	256 (>512)	512 (>512)	512 (>512)	32 (64)
*E. coli* DSM 8579	256 (>512)	512 (>512)	256 (512)	256 (>512)	512 (>512)	32 (32)
*S. enterica* DSM 9386	256 (>512)	512 (>512)	256 (512)	256 (>512)	512 (>512)	32 (32)
*P. hauseri* DSM 30118	256 (>512)	512 (512)	256 (512)	256 (>512)	256 (>512)	32 (32)

### The exposure of gram-positive foodborne pathogens to resveratrol derivatives results in membrane damage and the release of intracellular material

**Figure 4:**
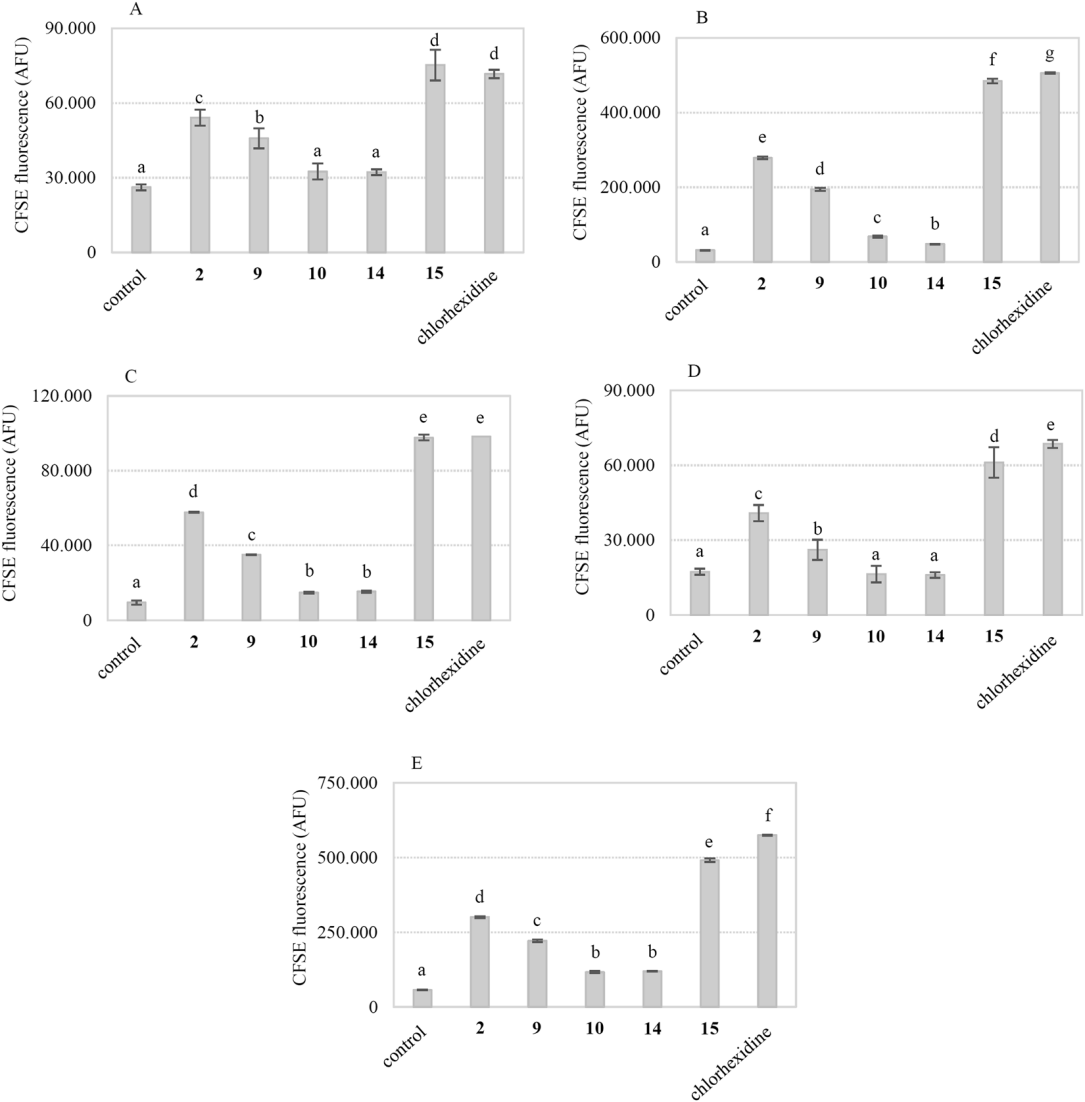
Leakage of cFSE fluorescence outside the Gram-positive cells due to membrane damages after exposure to 100 µg/mL of each compounds for 30 min at 30‒37 °C. (**A**) *B. cereus* DSM 9378. (**B**) *S. aureus* ATCC 25923. (**C**) *L. monocytogenes* Scott A. (**D**) *E. faecium* DSM 20477. (**E**) *E. faecalis* DSM 20478. Data are means of three replicates; standard deviation is shown as error bars. In the same chart, different letters indicate statistically significant differences between groups (p < 0.001).

Based on previous studies, we hypothesized that hydroxyl groups on the structure of polyphenolic compounds can interact with the bacterial cell membrane^30,31^. Thus, we investigated whether cell exposure to the selected compounds may result in membrane damage. Gram-positive bacterial cells were stained with the membrane-permeable dye cFDASE. Once inside the cell, cFDASE is cleaved by intracellular esterases, and the resultant fluorescent 5(6)-carboxyfluorescein succinimidyl ester (cFSE) molecules conjugate to the aliphatic amines of the intracellular proteins. The cFSE-labeled starving cells were then exposed to 100 µg/mL compounds **2**, **9**, **10**, **14**, **15** and chlorhexidine for 30 min at 30–37 °C. After incubation, the leakage of cFSE fluorescence outside the cells, as a result of membrane damage, was monitored by a fluorometer (Fig. 4)^32,33^. Pterostilbene **2**, its regioisomer **9**, and dimer **15** were able to determine a significant (P < 0.001) release of cFSE in all gram-positive strains tested, with **15** and chlorhexidine being the most active compounds. This evidence implies that the mechanism of action of these molecules involves the disruption of the phospholipid bilayer as for chlorhexidine (Fig. 4), which is known to target the plasma membrane of bacteria^29^. Conversely, the release of cFSE in the presence of **10** and **14** was comparable to or slightly higher than the control cells. Due to its ability to efficiently disrupt the cellular membrane, compound **15** was selected for further investigation.

### Effects of acute exposure of *L. monocytogenes* ScottA to compound 15 and chlorhexidine

**Figure 5:**
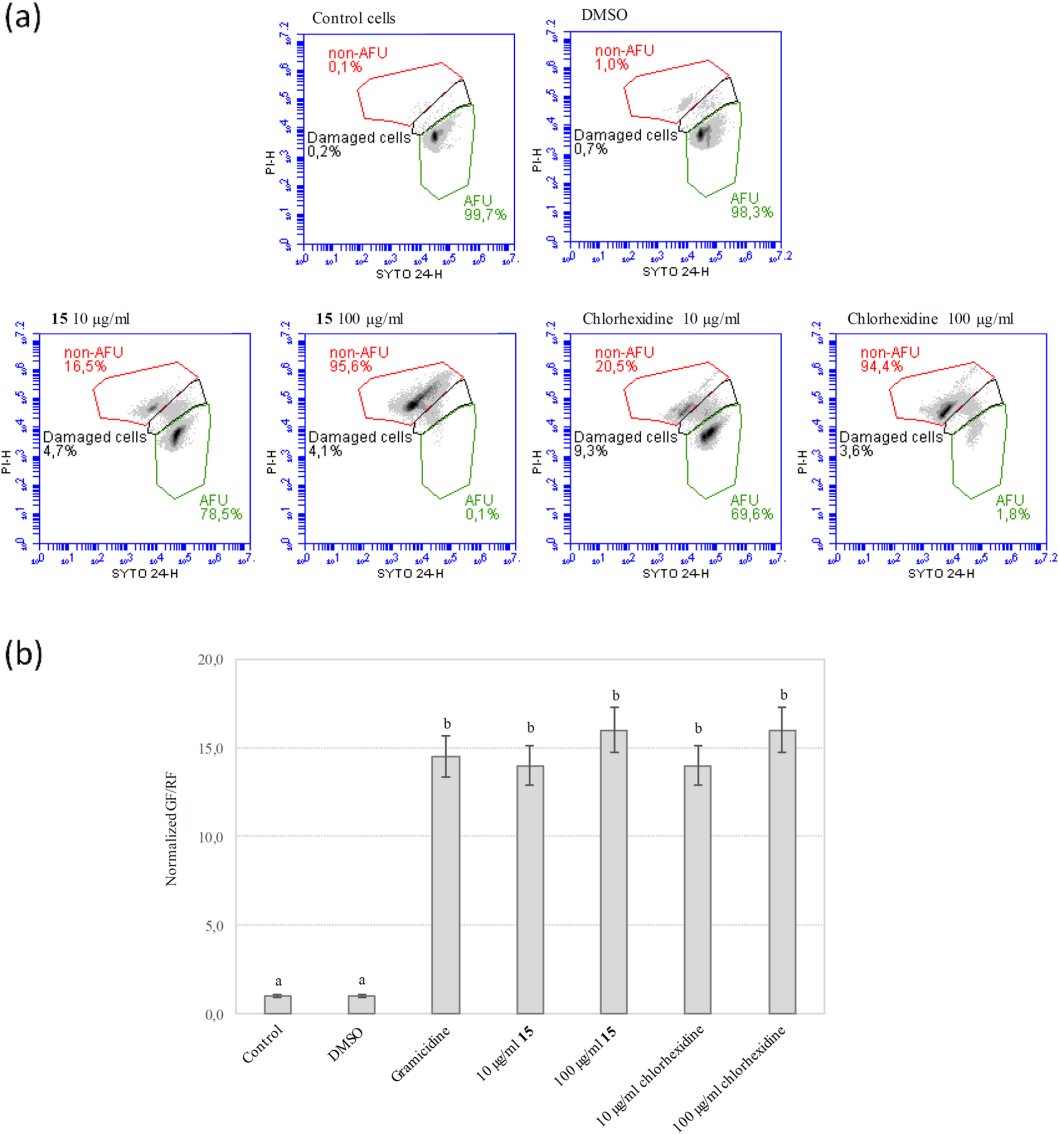
(**a**) Dot plot of cell viability determined by flow cytometry. Cell suspensions were stained with SYTO™ 24 and PI just after exposure to either **15** or chlorhexidine, for 30 min at 37 °C. Green gate: AFU (considered as live cells); dark gate: damaged cells; red gate: non-AFU (considered as dead cells). Inoculum was 9.0 ± 0.20 log_10_ AFU *L. monocytogenes* cells. Control cells were incubated in PBS; cells exposed to DMSO served as treatment control. (**b**) Effect of **15** and chlorhexidine exposure on membrane potential of *L. monocytogenes* ScottA cells. The membrane potential is reported as normalized green/red fluorescence (GF/RF). Fluorescence was measured by flow cytometry and GF/RF ratio considers the fluorescence of 30000 events. Gramicidin A used as standard molecule inducing membrane depolarization. Data are means of three replicates; standard deviation is shown as error bars. In the same chart, different letters indicate statistically significant differences between groups (p < 0.001).

Starving *L. monocytogenes* ScottA cells were exposed for 30 min at 37 °C to different concentrations (10 or 100 µg/mL) of either compound **15** or chlorhexidine. Then, cell viability, culturability and the effect on the membrane potential were assessed. The results obtained (Fig. 5) revealed that exposure to either compound **15** or chlorhexidine at the highest concentration (100 µg/mL) was sufficient to reduce cell viability by over 95%. When the lowest concentration (10 µg/mL) was tested, the decrease of the viable population (as AFU) was 22% and 30% in the presence of compound **15** or chlorhexidine, respectively (Fig. 5a).

Although the viability of the cells was only slightly affected by the presence of **15** (10 µg/mL, log_10_ CFU/mL 8.89 ± 0.11) or chlorhexidine (10 µg/mL, log_10_ CFU/mL 8.83 ± 0.12), the cell culturability after the same exposure was approximately 2.5-log lower than expected: 6.42 ± 0.28 and 6.60 ± 0.35 in the presence of **15** or chlorhexidine, respectively (Table S1). Conversely, no culturable cells were detectable after exposure to the higher concentration (100 µg/mL) of the two compounds, although the viability was not completely depleted (Table S1).

To better understand the mode of action of compound **15** on *L. monocytogenes* ScottA cells, we determined the cell membrane potential of the exposed cells that were further stained with DiOC_2_^3^. The observed decrease in red fluorescence indicated the disruption of the membrane potential that hampered the internalization of DiOC_2_; similar results were obtained in the presence of chlorhexidine and gramicidin A, an antimicrobial peptide known to induce dissipation of the electrochemical gradient across the cell membrane (Fig. 5b)^34^. Concurrently, the green fluorescence/red fluorescence ratio (GF/RF) of cells exposed to either **15** or chlorhexidine increased by approximately 15-fold, indicating complete cell membrane potential depletion irrespective of the antimicrobial concentration. No statistically significant differences were found between cells maintained in PBS or exposed to DMSO (p < 0.001); thus, the concentration of the solvent was not toxic to the cells, and the effects on the cell membrane were due to compound **15** and chlorhexidine.

### TEM analysis of *L. monocytogenes* after exposure to compound 15 and chlorhexidine

**Figure 6:**
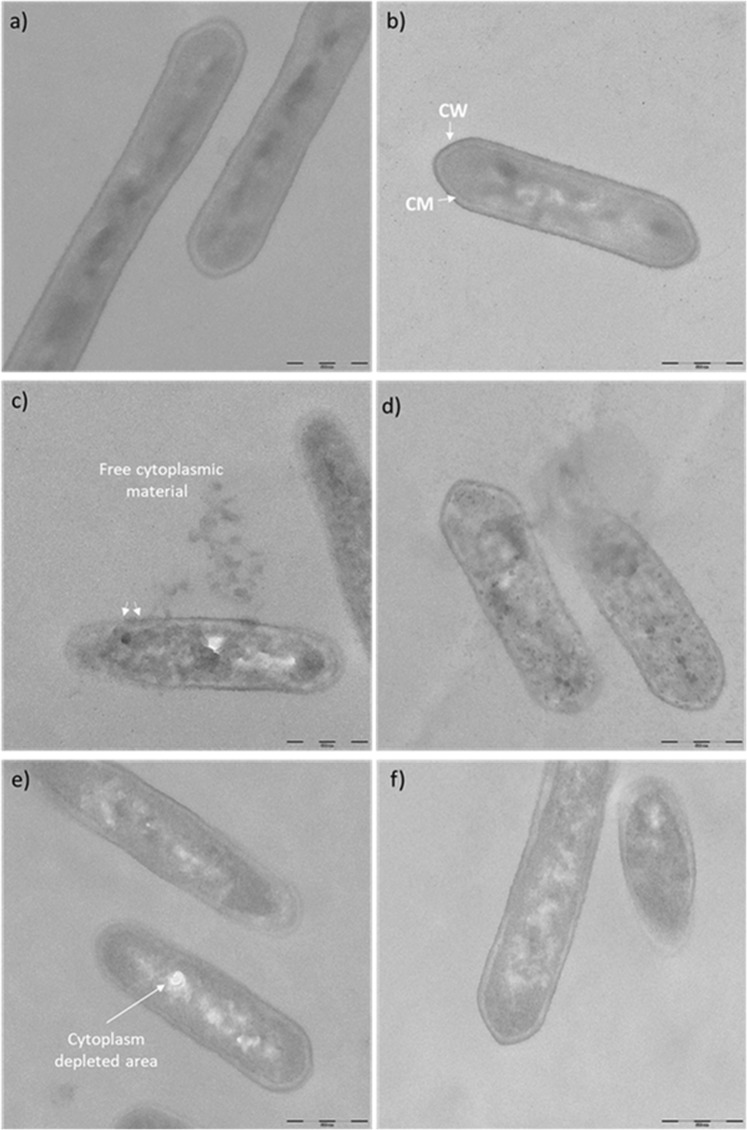
TEM micrographs of *L. monocytogenes* Scott A suspended in either PBS (**a**) or exposed to DMSO (**b**), 15 (**c**,**d**) or chlorhexidine (**e**,**f**). Intact cell wall (CW) and cell membrane (CM) were present in control samples (**a**,**b**). Cell wall or cell membrane interruptions (arrows) were visible in 15 exposed cells (**c**). Bar is 500 nm.

We considered achieving direct evidence of cell membrane damage to be conclusive in our study. Thus, the fine structure of *L. monocytogenes* cells exposed to **15** and chlorhexidine was investigated by transmission electron microscopy (TEM) of ultrathin sections of resin-embedded samples. Untreated cells, either in PBS or DMSO (controls), showed an evenly distributed cytoplasm within a continuous and smooth cell wall and cell membrane (Fig. 6a,b), whereas important modifications were observed after cell exposure to **15** (100 µg/mL) (Fig. 6c,d). The cytoplasm within the cell lost its even distribution, and several clumps appeared instead, with few spots likely corresponding to cytoplasm-depleted areas. This pattern was common to all treated cells. In addition, free cytoplasm was observed in the proximity of the cells that were most deeply damaged by **15**, as revealed by interruptions in either the cell wall or the cell membrane. Black spots were also visible within a few cells, possibly due to more extensive cytoplasm clumping or fixative accumulation (Fig. 6d). Interestingly, chlorhexidine caused only subtler damage to *L. monocytogenes* when tested at the same concentration (Fig. 6e,f). Indeed, no ruptures were found at the wall level but an increased roughness compared to controls, with accordingly no cytoplasmic material leakage out of the cells. The characteristic feature of cell damage caused by chlorhexidine is the presence of defined spots lacking cytoplasm within the cell.

## Discussion

The survival and growth of harmful microorganisms in food have been considered the primary causes of food-borne diseases. In recent years, the emergence of bacterial resistance to common antimicrobials coupled with the increasing negative awareness of consumers on synthetic preservatives has prompted researchers to discover novel natural and nature-derived preservatives. Natural products and their derivatives have been recognized for many years as a significant source of “privileged structures” in the development of bioactive molecules. In particular, phenolic compounds extracted from plants have been extensively screened due to their antimicrobial activities against a broad range of food poisoning microorganisms. Stilbenoids are an intriguing structural class of natural polyphenols characterized by a diverse array of biological and potential therapeutic properties^13^. The antimicrobial effects of resveratrol **1** and its dimethylated analog pterostilbene **2** are well documented, and multiple studies have suggested that they may have numerous preventive and therapeutic properties in a vast range of human diseases^15^.

Based on the many promising reports on the antimicrobial activity of resveratrol and pterostilbene, we decided to expand the investigation to a series of resveratrol-derived compounds characterized by different shapes, geometries and patterns of substitution to shed light on the SAR of this class of compounds.

To this aim, we prepared a small collection of resveratrol-derived monomers (**3**–**9**) and dimers (**10**–**15**) to be screened together with resveratrol **1** and pterostilbene **2**. Initially, the compounds were tested against representative gram-positive (*S. aureus* ATCC 25923) and gram-negative (*P. aeruginosa* ATCC 27853) bacteria (Table 1). The results confirmed that pterostilbene **2** was endowed with activity greater than that of resveratrol **1** against *S. aureus*, which is in accordance with already reported data^13^. In previous studies, resveratrol was demonstrated to possess antimicrobial activity against foodborne pathogens with a very high heterogeneity in MIC values ranging from 16.5 to 350 µg/mL for gram-positive bacteria and from 0.625 to 521 µg/mL for gram-negative bacteria, depending on the tested strain and experimental conditions (inoculum size, medium)^2,35,36^. The reference strains used in the first screening of our work revealed low sensitivity towards resveratrol **1** (≥512 µg/mL), with MIC values close to the indicated ranges. Conversely, higher antimicrobial activity was reported for pterostilbene **2**, confirming its greater sensitivity for gram-positive strains (25 µg/mL) rather than gram-negative strains (>100 µg/mL)^35^.

Moreover, out of the 9 monomers tested, only pterostilbene **2** and its regioisomer **9** were active. Importantly, among the dimers, compounds **10**, **14** and **15** showed considerable activity (MIC values of 2–16 µg/mL) against *S. aureus*. In general, both monomers and dimers were considerably less active against *P. aeruginosa*. The most promising molecules **2**, **9**, **10**, **14** and **15** were selected to be tested against a wider panel of microorganisms, including five species of gram-positive and four species of gram-negative bacteria. Our results showed that pterostilbene **2** has bacteriostatic activity at low concentrations (MIC 4–64 µg/mL), but higher concentrations were required for bactericidal effects on gram-positive bacteria (MBC 128–512 µg/mL). Analogous results were obtained for compounds **9**, **10** and **14** (MIC 4–128 µg/mL; MBC 128 to >512 µg/mL). According to the literature, molecules **9** and **10** have already demonstrated antimicrobial activity, with MIC values of 7–56 µg/mL in gram-positive foodborne pathogens^3^. The most active molecule was **15**, with MIC and MBC values of 1–4 and 16–64 µg/mL, respectively. None of the molecules were found to be active against gram-negative bacteria except at high concentrations (128–512 µg/mL) (Table 2). Indeed, there are numerous studies in the literature reporting that polyphenolic compounds are less effective against gram-negative bacteria, likely because of the outer complex membrane linked to the cell wall consisting of a rigid polysaccharide leaflet^37,38^. The lipopolysaccharide envelope can slow down the passage of phytochemicals and cytotoxic compounds^30^. In addition, active efflux of antimicrobials in gram-negative bacteria is performed by double-membrane spanning efflux systems with unusually broad substrate specificity, allowing the extrusion of substrates across the entire cell envelope^39^.

In an attempt to elucidate the mode of action of the synthesized stilbenoids, we investigated the changes in surface characteristic parameters of the exposed cells as well as alterations to their morphology and ultrastructure. Indeed, it is well documented that the accumulation of phenolic compounds in the lipid bilayer causes alterations in the membrane structure and could accelerate the extensive leakage of intracellular constituents, eventually leading to cell death^31^. Therefore, we assessed the damage to the gram-positive bacterial cell membrane after 30 min of exposure to the antimicrobials by measuring the efflux of the cFSE fluorescent molecule^33^. Among the tested compounds, dimer **15** was the most efficient at inducing cFSE leakage, whereas monomers **2** and **9** showed less significant cell membrane damage. We tried to rationalize the obtained results in light of the physicochemical properties of our compounds (Table S2), calculating the topological polar surface area, logP and several descriptors of the shape and geometry of the molecules. In this respect, it has been reported that the relative position of the OH groups on the phenolic nucleus influences the antibacterial efficacy of phenolic compounds^30,40^.

The obtained data suggested that the propensity to enter the membrane bilayer and interact with the phospholipids and proteins depends on an appropriate balance between lipophilicity and the presence of correctly oriented functional groups that are capable of establishing interactions with the main components of the cell membrane.

Starting from these considerations and based on the observed biological profile of dimer **15**, a further investigation on its mechanism of action was envisaged. *L. monocytogenes* ScottA was selected as a representative foodborne gram-positive bacterium to evaluate the effect of **15** on cell viability, culturability and membrane potential due to its scientific interest and ongoing effort to counteract its spread and virulence in the food chain. Interestingly, exposure of *L. monocytogenes* ScottA cells to **15** (10–100 µg/mL) induced depolarization of the cell membrane, an early indication of injury in bacteria, irrespective of the antimicrobial concentration^30,31,41^. In addition, exposure to a concentration of 100 µg/mL **15** induced the disruption of cell membrane integrity (Figs. 5 and 6). Moreover, acute exposure to **15** for a short period of time resulted in the loss of cell culturability, although viability was not completely depleted (Table S1). This discrepancy between culturability and viability could be explained by the induction of the viable-but-not-culturable (VBNC) state, usually attributed to environmental stress conditions, such as starvation, low temperature and antimicrobial exposure. Indeed, this state increases the ability of a bacterial population to survive under harsh conditions and supports the survival of foodborne pathogens in food processing plants. However, it is still not clear how VBNC cells can resuscitate back to the actively metabolizing state^42^. The data reported above on cell membrane disruption were further confirmed by TEM analysis. Exposure to **15** (100 µg/mL) caused irreversible damage to the cell membrane and coagulation of the cell content. Severe morphological modifications appeared in the cell wall and membrane, which led to the leakage of intracellular dense materials. In addition, the cell membrane surface was rougher in the treated *L. monocytogenes* cells than in the control cells, with pores and blebs appearing on the membrane surface and leakage of cytoplasmic materials (Fig. 6c,d).

Overall, the collected evidence indicates that the cell membrane (integrity and membrane-related processes, such as the proton gradient) represents the primary target of **15** (Fig. 5b), as previously demonstrated for other polyphenols^19^. Nevertheless, **15** might target other parts of the bacterial cell, and its antimicrobial activity could be ascribed to more than one specific mechanism. In this regard, further studies are required to understand the detailed mode of action of the selected compounds.

## Conclusions

Herein, we report our efforts to systematically study the antibacterial activity and mechanism of action of a set of resveratrol-derived stilbenoids, screened as single molecules against nine foodborne pathogens.The results showed that monomers **2** and **9** and dimers **10**, **14** and **15** were endowed with significant antibacterial activity against gram-positive bacteria, with dehydro-*δ*-viniferin **15** being the most potent molecule of the series. It was verified that the activity of **15** against *L. monocytogenes* was achieved by damaging the cytoplasmic membrane with significant membrane depolarization, a loss of membrane integrity, and severe morphological changes. To the best of our knowledge, this is the first report unveiling the membrane depolarization as the initial event in the antibacterial activity.

However, most likely, the bactericidal activity is not attributable exclusively to one specific mechanism, and additional targets may exist in the cell. In conclusion, the properties evidenced in our study for the resveratrol derivatives supported their potential applications as both antimicrobial agents against food-borne pathogens and antibiotic adjuvants^43^. Combination studies of the most active derivatives (**10**, **14** and **15**) with known antibiotics hold promise for future investigation^19,44^. Currently, to understand whether structural modifications can be exploited in the development of more potent congeners, the synthesis of additional analogs and simplified derivatives of dimers **10**, **14** and **15** is underway.

## Methods

### Chemical synthesis

Procedures for the synthesis, isolation, and characterization data for the various stilbenoid derivatives and intermediates are detailed in the Supplementary Materials. All synthesized derivatives were stored in dry form at −20 °C and dissolved in DMSO at a final concentration of 4.096 mg/mL prior to use in biological assays.

### Evaluation of the minimal inhibitory concentration (MIC) and minimal bactericidal concentration (MBC) of polyphenols against gram-positive and gram-negative bacteria

The molecules listed in Tables 1 and 2 were assayed for their antibacterial activity against both gram-positive and gram-negative foodborne pathogens. Among gram-positive bacteria, we selected *Listeria monocytogenes* Scott A, *Staphylococcus aureus* ATCC 25923, *Enterococcus faecium* DSM 20477, *Enterococcus faecalis* DSM 20478, and *Bacillus cereus* DSM 9378. Among the gram-negative bacteria, we considered *Pseudomonas aeruginosa* ATCC 27853, *Escherichia coli* DSM 682, *E. coli* DSM 8579, *Salmonella enterica* DSM 9386, and *Proteus hauseri* DSM 30118. The antibacterial activity was determined using the standard microdilution method for drug susceptibility testing^45^. The strains were cultivated aerobically in tryptic soy broth (TSB, Sigma Aldrich, Italy) for 24 h at 30–37 °C, except *E. faecium* DSM 20477 and *E. faecalis* DSM 20478, which were cultivated in microaerophilic conditions for 24 h at 30 °C. Briefly, the test was carried out in 96-well plates in a final volume of 200 µl in the presence of 10 increasing concentrations of each antimicrobial, ranging from 1 up to 512 µg/ml. The molecules were resuspended in dimethyl sulfoxide (DMSO, Sigma Aldrich, Italy) at a concentration of 4.096 mg/mL and then diluted 1:2 in sterile broth. The inoculum was prepared by diluting the cell suspension from an overnight culture in sterile TSB to obtain a turbidity equivalent to the McFarland 0.5 standard. Then, plates were incubated for 24 h at 30–37 °C. All plates included at least one well as a positive growth control (TSB with and without DMSO 12.5% v/v and the inoculum) and a negative growth control (TSB without inoculum), to exclude any microbial contamination^45^. As reference biocide, we selected chlorhexidine for its broad spectrum of activity against Gram positive and Gram negative bacteria and its known ability to disrupt the bacterial cell membrane. The MIC is the lowest concentration of the antimicrobial that completely inhibits growth. In addition, we determined the MBC for each molecule tested. After shaking the 96-well plate to resuspend the cell pellet, MBC determination was performed by subculturing 10 μL from each well where no visible microbial growth occurred. After 48 h of incubation, the antimicrobial dilutions yielding three colonies or less were scored as the MBC for the starting inocula of 10^5^ CFU/mL. The experiments were performed in triplicate. The MIC and MBC experiments were performed according to the CLSI (Clinical and Laboratory Standards Institute) methods for dilution antimicrobial susceptibility tests for aerobic bacteria (approved standard, Wayne, PA, USA: CLSI; 2009).

### Measurement of cFSE fluorescence cell leakage

To evaluate whether membrane damage was linked to cell leakage of intracellular components, microbial cells from an overnight culture were washed and diluted in sterile filtered (0.22 μm) phosphate-buffered saline (PBS) (NaCl 8 g/L; KCl 0.2 g/L; Na_2_HPO_4_ 1.44 g/L; KH_2_PO_4_ 0.24 g/L; pH 7.4) to a final concentration of 3 × 10^9^ cells per mL. The cell suspension was supplemented with 4 μM cFDASE (Sigma-Aldrich), which is a precursor molecule of cFSE^32^. Then, the cell suspension was exposed to each antimicrobial (100 μg/mL) or to chlorhexidine (100 μg/mL) (Sigma-Aldrich) at 30–37 °C (according to the optimum temperature of growth). As a control, the cFSE-labeled cell suspension was also exposed to a volume of DMSO equal to that used for all tested molecules. After 30 min of incubation, 1 mL of sample was used to measure cFSE fluorescence cell leakage. Briefly, the sample was centrifuged (13000 rpm, 2 min), and the cell-free supernatant was transferred to a 96-microtiter plate for the measurement of cFSE fluorescence in a Victor 3 fluorometer (PerkinElmer)^32,33^. The fluorescence data were calculated as the average of three independent assays and expressed as arbitrary units of fluorescence ± standard deviation.

### Flow cytometry to assess the viability and membrane potential of *L. monocytogenes* ScottA after exposure to compound 15 and chlorhexidine

A cell suspension in PBS (3 × 10^9^ cells per mL) from an overnight culture of *L. monocytogenes* ScottA was exposed to 10 or 100 μg/mL compound **15** or chlorhexidine for 30 min at 37 °C. As a control, we maintained cells in PBS with or without 2.4% DMSO (v/v) to simulate the same amount of DMSO used when the cells were treated with antimicrobials. Then, the viability of the cells was evaluated by flow cytometry with double staining with 20 μM SYTO^TM^ 24 (Thermo Scientific, Italy) and 2 μM propidium iodide (Sigma, Italy), according to ISO 19344 (2015). The effect of the treatment on the membrane potential was evaluated by staining cells with 3,3′-dimethyloxacarbocyanine iodide (DiOC_2_). Briefly, after incubation, the cells were stained with 30 µM DiOC_2_ (ThermoFisher Scientific, Milan, Italy) at 37 °C for 15 min in the dark. As a positive control, cells were previously incubated with 100 µM gramicidin A, an exogenous ion channel forming agent, inducing a decrease in the membrane potential and depolarization of the cells^34^. The dye DiOC_2_ has green fluorescence, and a redshift occurs when the dye self-associates in the cytosol when the membrane potential is large. A decrease in membrane potential leads to an increase in green fluorescence and a decrease in the ratio of green/red fluorescence^3^.

Stained samples were analyzed by a C6 Plus flow cytometer (BD Biosciences, Milan Italy) with thresholds FSC-H 4000 and SSC-H 1000. Green and red fluorescence were detected in the FL1 (excitation 488 nm, emission filter 530/30) and FL3 (excitation 488 nm, emission filter 670 LP) channels, respectively. In addition, to evaluate cell culturability, samples were subjected to a standard plate count on TSA (Sigma, Italy). Plates were incubated at 37 °C for 24–48 h under aerobic conditions.

### Transmission electron microscopy (TEM)

Cell suspensions of *L. monocytogenes* Scott A (3 × 10^9^ cells/mL) in PBS (0.1 M, pH 7.2, control) or exposed to DMSO (control), chlorhexidine (10 or 100 µg/mL) and Lux105 (10 or 100 µg/mL) were investigated at the ultrastructural level by TEM^46^. After 30 min incubation at 37 °C, cell suspensions were centrifuged at 10,000 × g for 10 min, and the pellets were fixed at 4 °C for 2 h with a solution containing 2% glutaraldehyde and 2% paraformaldehyde in 0.1 M sodium cacodylate solution buffered at pH 7.2 (Agar Scientific, Stansted, UK). Fixed cells were washed twice with sodium cacodylate solution and then suspended in 100 µL of low-temperature gelling agarose (2% w/v in water, melted at 35–40 °C) (VWR, Milan, Italy). The suspension was layered onto a microscope slide, allowed to set and then cut into 1 mm^3^ cubes. The cubes were incubated in the fixative solution for 1 h at 4 °C, then washed twice with sodium cacodylate buffer and postfixed in osmium tetroxide (EMS, Hatfield, USA) (1% in water, w/v) for 2 h. Samples were dehydrated in a series of ethanol solutions for 15 min (25, 50, 75, 90, 95 and 100%), embedded in Spurr resin (EMS, Hatfield, USA), and finally cured at 60 °C for 24 h. Ultrathin sections, 60 nm thick, were cut and stained with uranyl acetate and lead citrate (EMS, Hatfield, USA), both 0.2% in water (w/v). Sections were examined with a LEO912ab transmission electron microscope (Zeiss, Germany) at 100 kV, and digital images were acquired with an Esivision CCD-BM/1k system.

## Supplementary information

Supplementary Information
