# Supplementary material for: Antimicrobial activity of resveratrol-derived monomers and dimers against foodborne pathogens
Source: Sci Rep. 2019 Dec 20;9:19525. doi: 10.1038/s41598-019-55975-1 (PMC6925292; doi:10.1038/s41598-019-55975-1)
Supplement: Supplementary file 1 — Supplementary Information [file 41598_2019_55975_MOESM1_ESM.docx]

**Antimicrobial activity of resveratrol-derived monomers and dimers against foodborne pathogens**

Luce M. Mattio^1^, Sabrina Dallavalle^1^, Loana Musso^1^, Rossella Filardi^1^, Laura Franzetti^1^, Luisa Pellegrino^1^, Paolo D’Incecco^1^, Diego Mora^1^, Andrea Pinto^1*^, Stefania Arioli^1*^

^1^Department of Food, Environmental and Nutritional Sciences (DeFENS), University of Milan, Via Celoria 2, 20133 Milan, Italy. *email: stefania.arioli@unimi.it; andrea.pinto@unimi.it

**Supporting information**

**Table of contents**

**1) Tables**

Supplementary Table S1 S2

Supplementary Table S2 S2

**2) Synthesis**

1.1 General information S3

1.2 Experimental procedures S4

1.3 References S14

**1) Tables**

| **Compound** | **Culturability**  **as log_10_ CFU/ml** | **Viability**  **as log_10_ AFU/ml** |
| --- | --- | --- |
| Control | 8.98 ± 0.31 | 9.15 ± 0.05 |
| DMSO | 9.08 ± 0.26 | 9.19 ± 0.10 |
| **15** 10 μg/mL | 6.42 ± 0.28 | 8.89 ± 0.11 |
| **15** 100 μg/mL | < 1 | 6.01 ± 0.15 |
| chlorhexidine 10 μg/mL | 6.60 ± 0.35 | 8.83 ± 0.12 |
| chlorhexidine 100 μg/mL | < 1 | 7.39 ± 0.08 |

**Table S1.** Confronting culturability (as log_10_ CFU/ml) and viability (as log_10_ AFU/ml) of *L. monocytogenes* ScottA cells exposed to **15** or chlorhexidine.

| **cpd** | **Topological polar surface area Å^2^** | **Strongest acidic pKa** | **logP** | **logD** | **Minimum projection area Å^2^** | **Maximum projection area Å^2^** | **Solvent accessible surface area Å^2^** |
| --- | --- | --- | --- | --- | --- | --- | --- |
| **1** | 60.69 | 8.49 | 3.40 | 3.37 | 29.68 | 78.08 | 419.53 |
| **2** | 38.69 | 9.00 | 3.69 | 3.68 | 39.57 | 88.22 | 532.81 |
| **9** | 38.69 | 8.88 | 3.69 | 3.68 | 38.99 | 89.06 | 533.15 |
| **7** | 121.38 | 9.07 | 5.31 | 5.31 | 73.59 | 113.90 | 589.24 |
| **8** | 66.38 | 9.47 | 6.54 | 6.54 | 84.55 | 144.05 | 898.40 |
| **10** | 110.38 | 8.50 | 5.96 | 5.93 | 75.63 | 125.61 | 676.71 |
| **11** | 110.38 | 8.51 | 5.96 | 5.93 | 80.57 | 119.41 | 628.00 |
| **14** | 114.29 | 7.91 | 6.17 | 6.05 | 60.19 | 127.71 | 621.49 |
| **15** | 114.29 | 8.43 | 6.17 | 6.13 | 59.13 | 129.20 | 674.77 |

**Table S2.** Structural properties and geometries of compounds **1**-**15**. Values were calculated from [www.chemicalize.org](http://www.chemicalize.org).

**2) Synthesis**

**1.1 General information**

All reagents and solvents were reagent grade or were purified by standard methods before use. Unless otherwise specified, chemicals were from Sigma-Aldrich (Milan, Italy), as were the *Candida antarctica* lipase and the horseradish peroxidase used for some of the synthetic approaches. All reagents and solvents were reagent grade or were purified by standard methods before use. Melting points were determined on a model B-540 Büchi apparatus and are uncorrected. Optical rotation determinations were carried out using a Jasco P-1010 spectropolarimeter (Jasco Europe, Cremella, Italy), coupled with a Haake N3-B thermostat. NMR data were acquired using a Varian Mercury-300 MHz spectrometer (Varian, Palo Alto, CA, USA). Chemical shifts (*δ* values) and coupling constants (*J* values) are given in ppm and Hz, respectively. All reactions requiring anhydrous conditions were performed under a positive nitrogen flow and all glassware were oven dried and/or flame dried. Isolation and purification of the compounds were performed by flash column chromatography on silica gel 60 (230-400 mesh). Analytical thin-layer chromatography (TLC) was conducted on TLC plates (silica gel 60 F254, aluminum foil). Compounds on TLC plates were detected under UV light at 254 and 365 nm or were revealed spraying with 10% phosphomolybdenic acid (PMA) in ethanol. Procedures for synthesis, isolation, and characterization data for the various stilbenoid derivatives are detailed in the following Experimental Procedures section, together with literature references.

**1.2 Experimental procedures**

**(*E*)-4-(3,5-dimethoxystyryl)benzene-1,2-diol (4)**

To a solution of **2** (50 mg, 0.19 mmol, 1 eq) in DMF (2.6 mL) at room temperature, under nitrogen, IBX (64 mg, 0.290 mmol, 1.5 eq) was added and the reaction was stirred for 90 min. Then, a solution of NaBH_4_ (18 mg, 0.49 mmol, 2.5 eq) in MeOH (0.5 mL) was added at 0 °C and the mixture was stirred for 5 min. The reaction was quenched with 2N HCl at 0 °C until pH 1-2. The aqueous phase was extracted with EtOAc (4 × 10 mL). The combined organic layers were washed with NaHCO_3_ 5% solution (15 mL), a solution of water and brine 1:1 (3 × 15 mL), dried over Na_2_SO_4_ and concentrated under reduced pressure. The residue was purified by flash chromatography (FC) (Cyclohexane/EtOAc 7:3) to afford 33 mg of the desired product as a pale yellow solid. M.p.: 112-115 °C. Yield: 62%. R*_f_*: 0.28 (cyclohexane/EtOAc 7:3). ^1^H NMR (300 MHz, methanol-*d_4_*) δ 7.05 (d, *J* = 1.8 Hz, 1H), 6.98 (d, *J* = 16.2 Hz, 1H), 6.86 (dd, *J* = 1.8, 8.1 Hz, 1H), 6.83 (d, *J* = 16.2 Hz, 1H), 6.74 (d, *J* = 8.1 Hz, 1H), 6.64 (d, *J* = 1.8 Hz, 2H), 6.35 (t, *J* = 1.8 Hz, 1H), 3.80 (s, 6H). ^13^C NMR (75 MHz, acetone-*d_6_*) δ 161.0, 143.9, 143.8, 139.7, 130.9, 128.9, 127.1, 120.42, 115.7, 113.2, 104.6, 99.9, 55.7.^1^

**(*E*)-5-(4-acetoxystyryl)-1,3-phenylene diacetate** (**6**)

To the suspension of **1** (1 g, 4.38 mmol) in DCM (14 mL), Ac_2_O (1.5 mL, 15.26 mmol, 3.5 eq) and TEA (5 mL, 35.75 mmol, 8.2 eq) were added and the mixture was stirred at room temperature overnight. The reaction mixture was washed with NaHCO_3_ 5% solution and the aqueous phase was extracted with DCM (2 × 15 mL). The combined organic phases were washed with water (3 × 30 mL) and brine (30 mL), dried over Na_2_SO_4_ and concentrated under reduced pressure. The residue was purified by FC (cyclohexane/EtOAc 6:4). White solid. M.p.: 110-112 °C. Yield: 92%. R*_f_*: 0.40 (cyclohexane/EtOAc 7:3). ^1^H NMR: (300 MHz, chloroform-*d*) δ 7.50‒7.46 (m, 2H), 7.12 (d, *J* = 1.7 Hz, 2H), 7.11‒7.06 (m, 2H), 7.06 (d, *J* = 16.2 Hz, 1H), 6.96 (d, *J* = 16.2 Hz, 1H), 6.82 (t, *J* = 1.7 Hz, 1H), 2.31 (s, 9H). ^13^C NMR: (75 MHz, DMSO-*d_6_*) δ: 169.1, 168.9, 151.1, 150.2, 139.3, 134.2, 129.4, 127.6, 126.7, 122.1, 117.1, 114.9, 20.8, 20.7.^2^

**(*E*)-4-(3,5-dihydroxystyryl)benzene-1,2-diol (Piceatannol)** (**3**)

i) To a solution of **6** (400 mg, 1.13 mmol) in toluene (40 mL) and n-BuOH (1.6 mL, 17.5 mmol, 15.5 eq) at 40 °C, *Candida antarctica lipase* (400 mg) was added and the mixture was stirred (400 rpm) at the same temperature for 6 h. The reaction was cooled to room temperature, quenched by filtering off the enzyme and the filtrate was evaporated in vacuo. The residue was purified by FC (DCM/EtOAc 95:5) to give 281 mg of (*E*)-5-(4-hydroxystyryl)-1,3-phenylene diacetate as a white solid. M.p.: 146-147 °C. Yield: 81%. R*_f_*: 0.55 (DCM/EtOAc 95:5). ^1^H NMR: (300 MHz, acetone-*d*_6_) δ 7.52–7.43 (m, 2H), 7.23 (d, *J* = 16.2 Hz, 1H), 7.20 (d, *J* = 1.7 Hz, 2H), 7.04 (d, *J* = 16.2 Hz, 1H), 6.90–6.83 (m, 2H), 6.82 (t, *J* = 1.7 Hz, 1H), 2.28 (s, 6H). ^13^C NMR: (75 MHz, acetone-*d_6_*) δ 205.5, 168.6, 157.7, 151.7, 140.2, 130.4, 128.2, 123.9, 116.6, 115.6, 114.2, 20.1.

ii) To the solution of (*E*)-5-(4-hydroxystyryl)-1,3-phenylene diacetate (50 mg, 0.15 mmol) in DMF (2 mL) at room temperature, under nitrogen, IBX (64 mg, 0.29 mmol, 1.5 eq) was added and the reaction was stirred for 90 min. Then, a solution of NaBH_4_ (15 mg, 0.38 mmol, 2.5 eq) in MeOH (0.5 mL) was added at 0 °C and the mixture was stirred for 5 min. A chromatic change of the solution from red to yellow was observed. Then, the reaction was quenched with HCl 2N at 0 °C until pH 1-2. The aqueous phase was extracted with EtOAc (4 × 10 mL). The combined organic layers were washed with 5% NaHCO_3_ aqueous solution (15 mL) and a solution of water and brine 1:1 (3 × 15 mL), then dried over Na_2_SO_4_ and concentrated under reduced pressure. The residue was purified by FC (DCM/EtOAc 95:5) to afford 20 mg of the desired product as a yellow oil. Yield: 42%. R_f_: 0.38 (DCM/EtOAc 95:5). ^1^H NMR: (300 MHz, acetone-*d_6_*) δ 8.10 (brs, 2H), 7.19 (d, *J* = 1.7 Hz, 2H), 7.16 (d, *J* = 16.2 Hz, 1H), 7.13 (d, *J* = 1.8 Hz, 1H), 6.98 (d, *J* = 16.2 Hz, 1H), 6.97 (dd, *J* = 1.8, 8,1 Hz, 1H), 6.83 (d, *J* = 8,1 Hz, 1H), 6.81 (dt, *J* = 1.7 Hz, 1H), 2.28 (s, 6H). ^13^C NMR: (75 MHz, acetone-*d_6_*) δ 170.0, 151.2, 144.2, 140.4, 130.3, 129.7, 123.9, 120.1, 116.9, 115.3, 113.6, 113.1, 21.1.^3^

iii) To the solution of (*E*)-5-(3,4-dihydroxystyryl)-1,3-phenylene diacetate (93 mg, 0.28 mmol, 1 eq) in MeOH at room temperature, under nitrogen, hydrazine monohydrate (55 µL, 1.13 mmol, 4 eq) was added and the reaction was stirred for 20 min. Then, the solvent was evaporated, the residue was diluted with EtOAc and acidified with HCl 2N under nitrogen until pH 1-2. The aqueous phase was extracted with EtOAc (4 × 10 mL). The combined organic layers were washed with brine (20 mL), dried over Na_2_SO_4_ and concentrated under reduced pressure. The residue was purified by FC (DCM/MeOH 9:1) to afford 56 mg of the desired product as a white solid. M.p.: 228-229 °C. Yield: 81%. R*_f_*: 0.31 (DCM/MeOH 9:1). ^1^H NMR (300 MHz, methanol-*d_4_*) δ 7.97 (brs, 1H), 6.98 (d, *J* = 1.8 Hz, 1H), 6.88 (d, *J* = 16.5 Hz, 1H), 6.82 (dd, *J* = 8.1, 1.8 Hz, 1H), 6.74 (d, *J* = 16.5 Hz, 1H), 6.73 (d, *J* = 8.1 Hz, 1H), 6.44 (d, *J* = 1.8 Hz, 2H), 6.16 (t, *J* = 1.8 Hz, 1H). ^13^C NMR (75 MHz, acetone-*d_6_*) δ 158.6, 145.3, 145.2, 140.2, 130.0, 128.8, 126.2, 119.5, 115.7, 113.2, 105.2, 102.1.^4^

**(*E*)-1,3-dimethoxy-5-(4-methoxystyryl)benzene** (**5**)

To a solution of **1** (500 mg, 2.19 mmol) in dry acetone (10 mL), at room temperature, under nitrogen, was added K_2_CO_3_ (2.725 g, 19.7 mmol, 9 eq). After 10 min. stirring, CH_3_I (1.23 mL, 19.7 mmol, 9 eq) was added dropwise. The mixture was stirred overnight. Then the solvent was evaporated, the residue was quenched with a saturated solution of NH_4_Cl (15 mL) and H_2_O (10 mL). The aqueous phase was extracted with EtOAc (3 × 20 mL). The combined organic layers were washed with H_2_O (20 mL), brine (20 mL), dried over Na_2_SO_4_ and concentrated under reduced pressure. The residue was purified by FC (cyclohexane/EtOAc 8:2) to afford 485 mg of the desired product as a white solid. M.p.: 55-57 °C. Yield: 81%. R*_f_*: 0.45 (cyclohexane/AcOEt 8:2). ^1^H NMR (300 MHz, chloroform-*d*) δ 7.48–7.41 (m, 2H), 7.04 (d, *J* = 16.2 Hz, 1H), 6.94–6.86 (m, 3H), 6.65 (d, *J* = 2.2 Hz, 2H), 6.38 (t, *J* = 2.2 Hz, 1H), 3.83 (s, 9H). ^13^C NMR (100 MHz, chloroform-*d*) 161.0, 159.4, 139.7, 129.9, 128.7, 127.8, 126.6, 114.2, 104.4, 99.6, 55.3.^5^

**(*E*)-5-(4-methoxystyryl) benzene-1,3-diol (7) and (*E*)-3-(4-hydroxystyryl)-5-methoxyphenol (8)**

To a solution of resveratrol (**1**) (1 g, 4.88 mmol) in dry acetone (20 mL) at rt and under nitrogen, K_2_CO_3_ (1.2 g, 8.76 mmol, 2 eq) and CH_3_I (818 µl, 13.14 mmol, 3 eq) were added dropwise. The mixture was stirred overnight. Then, the solvent was evaporated, the residue diluted with EtOAc and washed with H_2_O. The aqueous phase was extracted with EtOAc (3 × 20 mL). The combined organic layers were washed with brine (20 mL), dried over Na_2_SO_4_ and concentrated under reduced pressure. The residue was purified by FC (DCM/ EtOAc 95:5) to obtain 75 mg of **8**, 102 mg of **7** and 340 mg of a mixture **9**+**2**.

Compound **7**: Yield: 15%. R_f_: 0.29 (DCM/EtOAc 92:8) M.p. 176-178 °C. ^1^H NMR (300 MHz, DMSO-*d*_6_) δ 9.19 (brs, 2H), 7.54–7.47 (m, 2H), 6.97 (d, *J* = 16.2, 1H), 6.94–6.88 (m, 2H), 6.87 (d, *J* = 16.2, 1H), 6.39 (d, *J* = 2.1 Hz, 2H), 6.11 (t, *J* = 2.1 Hz, 1H), 3.75 (s, 3H). ^13^C NMR (100 MHz, chloroform-*d*), δ: 161.2, 160.4, 141.6, 131.8, 129.6, 129.4, 128.3, 115.7, 116.6, 103.6, 56.4.^5^

Compound **8**: Yield 12%. R*_f_*: 0,41 (DCM/EtOAc 92:8) 117-118 °C; ^1^H NMR (300 MHz, DMSO-*d*_6_) δ: 7.42–7.35 (m, 2H), 7.02 (d, *J* = 16.3 Hz, 1H), 6.86 (d, *J* = 16.3 Hz, 1H), 6.77–6.71 (m, 2H), 6.55 (dd, *J* = 2.3, 1.4 Hz, 1H), 6.51 (dd, *J* = 2.3, 1.4 Hz, 1H), 6.20 (t, *J* = 2.3 Hz, 1H), 3.71 (s, 3H). ^13^C NMR (100 MHz, chloroform-*d*) δ 162.5, 159.7, 158.4, 141.3, 130.3, 129.8, 129.9, 128.9, 116.5, 106.6, 104.4, 101.4, 55.6.^6^

**(*E*)-4-[3,5-Bis(*tert*-butyldimethylsilyloxy)styryl](*tert*-butyldimethylsilyloxy) benzene (16)**

To a solution of **1** (500 mg, 2.19 mmol) in anhydrous DMF (15 mL) at −20 °C, under nitrogen, was added a solution of TBDMSCl (1.32 g, 8.76 mmol, 4 eq) in anhydrous DMF (5 mL) and DIPEA (1.5 mL, 8.76 mmol, 4 eq). The reaction mixture was allowed to warm to room temperature. After 16 h the resulting reaction mixture was diluted with EtOAc (70 mL) and washed with a 1:1 v/v solution 0,1 N HCl /brine (5 × 30 mL). The organic layer was dried over Na_2_SO_4_ and concentrated under reduced pressure. The residue was purified by FC (DCM/cyclohexane 3:7) to obtain 1.19 g of product as a white amorphous solid. Yield: 95%. R*_f_*: 0.7 (cyclohexane/DCM 8:2). ^1^H NMR: (300 MHz, chloroform-*d*) δ: 7.41–7.35 (m, 2H), 6.96 (d, *J* = 16.2 Hz, 1H), 6.83 (d, *J* = 16.2 Hz, 1H), 6.85–6.80 (m, 2H), 6.60 (d, *J* = 2.2 Hz, 2H), 6.24 (t, *J* = 2.2 Hz, 1H), 1.00 (s, 27H), 0.22 (s, 18H). ^13^C NMR (126 MHz, chloroform-*d*) δ 156.8, 155.6, 139.6, 130.8, 128.6, 127.8, 126.9, 120.5, 111.7, 111.4, 25.9, 25.8, 18.4, −4.2, −4.2.^7^

**(*E*)-3-(*tert*-butyldimethylsilyloxy)-5-(4-hydroxystyryl)phenol** (**17**)

To a solution of **16** (600 mg, 1.05 mmol) in dry THF (12 mL) was added a solution of potassium fluoride (61 mg, 1.05 mmol, 1.0 eq) in MeOH (12 mL) at −15 °C under nitrogen and the mixture was vigorously stirred for 6 h. The resulting reaction mixture was concentred under reduced pressure then diluted with AcOEt and washed with 50% brine (2 × 30 mL). The organic layer was dried over Na_2_SO_4_ and concentrated under reduced pressure. The resulting crude product was purified by FC (DCM/acetone 95:5) to afford 201 mg of product as a white solid. M.p.: 62-63 °C Yield: 56%. R*_f_*: 0.39 (DCM/acetone 95:5) ^1^H NMR (300 MHz, chloroform-*d*) δ: 7.43–7.38 (m, 2H), 6.97 (d, *J* = 16.3 Hz, 1H), 6.86–6.80 (m, 2H), 6.83 (d, *J* = 16.3 Hz, 1H), 6.59 (dd, *J* = 2.3, 1.4 Hz, 1H), 6.55 (dd, *J* = 2.3, 1.4 Hz, 1H), 6.25 (t, *J* = 2.3 Hz, 1H), 4.95 (brs, 1H), 4.81 (brs, 1H), 1.00 (s, 9H), 0.23 (s, 6H). ^13^C NMR (126 MHz, chloroform-*d*) δ 157.2, 156.7, 155.4, 140.0, 130.3, 128.8, 128.2, 126.4, 115.8, 111.3, 106.8, 106.5, 25.8, 18.4, −4.2.^7^

**(*E*)-3-methoxy-5-(4-methoxystyryl)phenol** (**9**)

To a solution of **17** (109 mg, 0.318 mmol) in dry acetone (1.5 mL) at room temperature, under nitrogen, was added K_2_CO_3_ (264 mg, 1.91 mmol, 6 eq). After 10 minutes stirring CH_3_I (118 µL, 1.91 mmol, 6 eq) was added dropwise. The solution was stirred overnight. Then, the solvent was evaporated, the residue was quenched with a saturated aqueous NH_4_Cl solution. The aqueous phase was extracted with EtOAc (3 × 10 mL). The combined organic layers were washed with H_2_O (20 mL) brine (20 mL) dried over Na_2_SO_4_ and concentrated under reduced pressure. The residue was purified by FC (Cyclohexane/DCM 7:3) to afford 45 mg of 3-(*tert*-butyldimethylsilyloxy)-4′,5-dimethoxystilbene as a colourless oil. Yield: 38%. R_f_: 0.31 (cyclohexane/DCM 7:3) ^1^H NMR (300 MHz, chloroform-*d*) δ: 7.49–7.42 (m, 2H), 7.02 (d, *J* = 16.3 Hz, 1H), 6.94–6.88 (m, 2H), 6.89 (d, *J* = 16.3 Hz, 1H), 6.68 (dd, *J* = 2.1, 1.4 Hz, 1H), 6.61 (dd, *J* = 2.1, 1.4 Hz, 1H), 6.32 (t, *J* = 2.1 Hz, 1H), 3.84 (s, 3H), 3.82 (s, 3H), 1.01 (s, 9H), 0.24 (s, 6H). ^13^C NMR (75.5 MHz, chloroform-*d*) δ 160.8, 159.4, 156.9, 139.6, 130.0, 128.5, 127.8, 126.6, 114.1, 110.9, 104.8, 55.3, 25.7, 14.1, −4.4.^8^

To a solution of 3-(*tert*-butyldimethylsilyloxy)-4′,5-dimethoxystilbene (28 mg, 0.0756 mmol) in dry MeOH (1 mL) was added a solution of potassium fluoride (26 mg, 0.453 mmol, 6 eq) in MeOH 2:1 THF (1.5 mL) dropwise at room temperature. The mixture was stirred under nitrogen overnight. The resulting reaction mixture was concentred under reduced pressure then diluted with EtOAc and washed with 50% brine (2 × 30 mL). The organic layer was dried over Na_2_SO_4_ and concentrated under reduced pressure. The resulting crude product was purified by FC (cyclohexane/ EtOAc 7:3) to obtain 19 mg of product as a white solid. M.p.: 115-117 °C. Yield: 100%. R*_f_*: 0.29 (cyclohexane/EtOAc 7:3) ^1^H NMR (300 MHz, chloroform-*d*) δ: 7.47–7.41 (m, 2H), 7.02 (d, *J* = 16.3 Hz, 1H), 6.94–6.88 (m, 2H), 6.87 (d, *J* = 16.3 Hz, 1H), 6.63 (dd, *J* = 2.1, 1.4 Hz, 1H), 6.58 (dd, *J* = 2.1, 1.4 Hz, 1H), 6.32 (t, *J* = 2.1 Hz, 1H), 3.84 (s, 3H), 3.82 (s, 3H).^13^C NMR (100 MHz, DMSO-*d_6_*) δ: 160.6, 159.0, 158.6, 139.3, 129.6, 128.0, 127.8, 126.4, 114.1, 106.0, 102.6, 100.6, 55.1, 55.0.^9^

(***E*)-5-((±)-6-hydroxy-2-(4-hydroxyphenyl)-4-(4-hydroxystyryl)-2,3-dihydrobenzofuran-3-yl)benzene-1,3-diol (*trans*-ε-viniferin)** (±)-**11**

To a solution of **1** (2 g, 8.76 mmol, 1 eq) in MeOH (20 mL) a solution of FeCl_3_.6H_2_O (2.39 g, 8.85 mmol, 1.01 eq) in water (20 mL) was added dropwise. The mixture was stirred at room temperature for 48 hours. MeOH was evaporated and the residue was diluted with water (30 mL) and extracted with EtOAc (4 × 50 mL). The combined organic layers were dried over anhydrous Na_2_SO_4_ and concentrated under reduced pressure. The residue was purified by FC (cyclohexane/acetone 3:2) and a mixture of (±)-**11** and *cis-*analogue was obtained. A further purification by reverse phase column chromatography (H_2_O/MeOH 2:3) gave 196 mg of (±)-**11** as a light green amorphous solid. Yield: 15%. R*_f_*: 0.30 (cyclohexane/acetone 3:2) or 0.42 (H_2_O/MeOH 2:3). ^1^H NMR: (300 MHz, Methanol-*d*_4_) δ 7.17–7.10 (m, 2H), 7.07–7.01 (m, 2H), 6.82 (d, *J* = 16.2, Hz, 1H), 6.78–6.73 (m, 2H), 6.67–6.62 (m, 2H), 6.63 (s, 1H), 6.56 (d, *J* = 16.2 Hz, 1H), 6.24 (d, *J* = 2.1 Hz, 1H), 6.20–6.14 (m, 3H), 5.36 (d, *J* = 6.6 Hz, 1H), 4.34 (d, *J* = 6.6 Hz, 1H). ^13^C NMR (100 MHz, methanol-*d*_4_) *δ*: 162.8, 160.1, 159.8, 159.7, 158.5, 158.4, 147.4, 137.0, 136.9, 133.9, 130.5, 130.4, 128.8, 128.2, 123.8, 123.7, 120.1, 116.4, 116.3, 107.5, 104.4, 102.2, 96.9, 94.9, 58.3.^6^

**(*E*)-5-((±)-6-acetoxy-2-(4-acetoxyphenyl)-4-(4-acetoxystyryl)-2,3-dihydrobenzofuran-3-yl)-1,3-phenylene diacetate** (±)-**18**

To a solution of (±)-**11** (140 mg, 0.31 mmol, 1 eq) in DMSO (0.8 mL) and DCM (19.8 mL) at rt, Ac_2_O (0.19 mL, 2 mmol, 6.6 eq) and TEA (0.63 mL, 4.56 mmol, 14.7 eq) were added and the mixture was stirred at room temperature overnight. Solvent was evaporated and the residue was diluted with EtOAc (10 mL) and washed with 5%NaHCO_3_ aqueous solution (10 mL). The aqueous phase was extracted with EtOAc (2 × 10 mL). The combined organic layers were washed with water (20 mL), dried over anhydrous Na_2_SO_4_ and concentrated under reduced pressure. The residue was purified by FC (cyclohexane/EtOAc 3:2) to afford 191 mg the desired product as a white sticky solid. Yield: 92%. R*_f_*: 0.35 (cyclohexane/EtOAc 3:2) ^1^H NMR: (300 MHz, chloroform-*d*) δ 7.37–7.31 (m, 2H), 7.21–7.15 (m, 2H), 7.14–7.07 (m, 2H), 7.02–6.96 (m, 2H), 6.95 (d, *J* = 2.2 Hz, 1H), 6.91 (t, *J* = 2.2 Hz, 1H), 6.88 (d, *J* = 16.2 Hz, 1H), 6.86 (d, *J* = 2.2 Hz, 1H), 6.65 (d, *J* = 2.2 Hz, 1H), 6.54 (d, *J* = 16.2 Hz, 1H), 5.60 (d, *J* = 6.9 Hz, 1H), 4.50 (d, *J* = 6.9 Hz, 1H), 2.34 (s, 3H), 2.31 (s, 3H), 2.28 (s, 3H), 2.27 (s, 6H). ^13^C NMR (100 MHz, chloroform-*d*) δ 69.6, 169.5, 169.5, 168.9, 161.1, 152.2, 151.8, 150.8, 150.5, 144.5, 138.2, 135.4, 134.5, 130.5, 127.9, 126.8, 124.2, 124.0, 122.1, 121.9, 118.7, 115.0, 110.8, 103.0, 92.8, 56.8, 21.4, 21.3, 21.2.^10^

**(*E*)-5-(6-hydroxy-2-(4-hydroxyphenyl)-4-(4-hydroxystyryl)benzofuran-3-yl)benzene-1,3-diol (Viniferifuran) 14**

i) To a solution of (±)-**18** (38 mg, 0.057 mmol, 1 eq) in DCM (5 mL) at r.t., was added DDQ (260 mg, 1.14 mmol, 20 eq). The mixture was stirred under reflux for 48 hours. The mixture was cooled to room temperature, then filtered on a celite pad and concentrated under reduced pressure. The residue was purified by FC (cyclohexane/EtOAc 7:3) to give 32 mg of (*E*)-5-(6-acetoxy-2-(4-acetoxyphenyl)-4-(4-acetoxystyryl)benzofuran-3-yl)-1,3-phenylene diacetate as a light brown sticky solid Yield: 84%. R*_f_*: 0.3 (cyclohexane/ EtOAc 3:2) ^1^H NMR (300 MHz, chloroform-*d*) δ 7.60–7.54 (m, 2H),7 .25 (d, *J* = 2.2 Hz, 1H), 7.24 (d, *J* = 2.2 Hz, 1H), 7.14–7.09 (m, 4H), 7.08–7.02 (m, 2H), 7.02–6.98 (m, 2H), 6.93 (d, *J* = 16.3 Hz, 1H), 6.87 (d, *J* = 16.3 Hz, 1H), 2.38 (s, 3H), 2.29 (m, 6H), 2.25 (s, 6H).

ii) To the suspension of (*E*)-5-(6-acetoxy-2-(4-acetoxyphenyl)-4-(4-acetoxystyryl)benzofuran-3-yl)-1,3-phenylene diacetate (32 mg, 0.048 mmol, 1 eq) in MeOH at 0 °C was added KOH 85% (32 mg, 0.48 mmol, 10 eq). The mixture was stirred at 0 °C for 90 min. and then acidified with 0.1 N HCl until pH 1. MeOH was evaporated and residue was diluted with water (5 mL), extracted with EtOAc (3 × 10 mL). The organic phase was washed with water (10 mL) and brine (10 mL), dried over anhydrous Na_2_SO_4_ and concentrated under reduced pressure. The residue was purified by FC (cyclohexane/acetone 1:1) to afford 12 mg of the desired product as a yellow amorphous solid. Yield: 55%. R*_f_*: 0.3 (cyclohexane/acetone 3:2) ^1^H NMR: (300 MHz, methanol-*d*_4_) δ 7.45–7.40 (m, 2H), 7.01–6.92 (m, 4H), 6.85 (d, *J* = 16.3 Hz, 1H), 6.81 (d, *J* = 2.0 Hz, 1H), 6.72–6.68 (m, 2H), 6.68–6.63 (m, 2H), 6.48 (t, *J* = 2.0 Hz, 1H), 6.41 (d, *J* = 2.0 Hz, 2H). ^13^C NMR: (100 MHz, methanol-*d*_4_) *δ* 160.7, 158.5, 158.2, 156.6, 156.4, 150.6, 138.7, 133.3, 130.7, 129.3, 128.8, 128.5, 123.9, 123.3, 122.5, 117.4, 116.4, 116.2, 110.2, 107.4, 103.1, 97.4.^11^

**(*E*)-5-((±)-2-(3-(3,5-dihydroxyphenyl)-2-(4-hydroxyphenyl)-2,3-dihydrobenzofuran-5-yl)vinyl)benzene-1,3-diol (δ-viniferin)** (±)-**10**

To a solution of **1** (1g, 4.38 mmol, 1 eq) in acetone (21.9 mL): citrate buffer pH 5 (21.9 mL) was added a solution of HRP (1.75 mL, 1 mg/mL aqueous solution) at 40 °C and the mixture was stirred for 30 minutes. Then H_2_O_2_ 30% (0.66 mL) was added to the solution and the mixture was stirred at the same temperature for 1h. The acetone was evaporated and the aqueous layer was extracted with EtOAc (3 × 50 mL). The combined organic layers were washed with brine, dried over anhydrous Na_2_SO_4_ and concentrated under reduced pressure. The residue was purified by flash column chromatography (DCM/MeOH 9:1) to give 473 mg of the desired product as a yellow amorphous solid. Yield: 49%. R*_f_*: 0.25 (DCM/MeOH 9:1). ^1^H NMR: (300 MHz, methanol-*d*_4_) δ 7.36 (dd, *J* = 8.1, 1.8 Hz, 1H), 7.20–7.14 (m, 2H), 7.18 (d, *J* = 1.8 Hz, 1H), 6.98 (d, *J* = 16.2 Hz, 1H), 6.85 (d, *J* = 8.1 Hz, 1H), 6.80–6.74 (m, 2H), 6.78 (d, *J* = 16.2 Hz, 1H), 6.43 (d, *J* = 2.2 Hz, 2H), 6.19 (t, *J* = 2.2 Hz, 1H), 6.14 (t, *J* = 2.2 Hz, 1H), 6.12 (d, *J* = 2.2 Hz, 2H), 5.38 (d, *J* = 8.4 Hz, 1H), 4.40 (d, *J* = 8.4 Hz, 1H). ^13^C NMR: (75 MHz, methanol-*d_4_)* δ 161.0, 159.0, 159.7, 158.8, 145.4, 141.2, 132.9, 132.4, 132.4, 129.4, 128.7, 127.5, 124.2, 116.3, 110.4, 107.8, 105.8, 102.7, 102.5, 94.9, 58.0.^12^

**(*E*)-5-((±)-2-(2-(4-acetoxyphenyl)-3-(3,5-diacetoxyphenyl)-2,3-dihydrobenzofuran-5-yl)vinyl)-1,3-phenylene diacetate** (±)-**19**

To a solution of (±)-**10** (100 mg, 0.22 mmol, 1 eq) in DMSO (1.5 mL) and DCM (12 mL) at r.t., Ac_2_O (0.14 mL, 1.45 mmol, 6.6 eq) and TEA (0.45 mL, 3.2 mmol, 14.7 eq) were added and the mixture was stirred at room temperature overnight. Solvent was evaporated and the residue was diluted with EtOAc (10 mL) and washed with 5% NaHCO_3_ aqueous solution (10 mL). The aqueous phase was extracted with EtOAc (2 × 10 mL). The combined organic layers were washed with water (20 mL), dried over anhydrous Na_2_SO_4_ and concentrated under reduced pressure. The residue was purified by FC (cyclohexane/ EtOAc 3:2) to afford 131 mg of the desired product as a white amorphous solid. Yield: 90% R*_f_*: 0.32 (cyclohexane/ EtOAc 3:2) ^1^H NMR: (300 MHz, chloroform-*d*) δ 7.39–7.31 (m, 3H), 7.22–7.18 (m, 1H), 7.13–7.08 (m, 2H), 7.07 (d, *J* = 2.1 Hz, 2H), 7.01 (d, *J* = 16.3 Hz, 1H), 6.97–6.92 (m, 3H), 6.91 (d, *J* = 16.3 Hz, 1H), 6.82 (d, *J* = 2.1 Hz, 2H), 6.78 (t, *J* = 2.1 Hz, 1H), 5.55 (d, *J* = 8.0 Hz, 1H), 4.54 (d, *J* = 8.0 Hz, 1H), 2.30 (s, 3H), 2.29 (s, 6H), 2.27 (s, 6H). ^13^C NMR: (75 MHz, chloroform-*d)* δ 169.3, 168.9, 168.8, 159.9, 151.4, 151.3, 150.7, 143.7, 139.9, 137.7, 130.7, 130.2, 129.7, 128.5, 126.9, 124.9, 123.4, 121.9, 118.7, 116.6, 114.7, 113.9, 110.0, 92.4, 57.3, 21.1.

**(*E*)-5-(2-(3-(3,5-dihydroxyphenyl)-2-(4-hydroxyphenyl)benzofuran-5-yl)vinyl)benzene-1,3-diol (15**)

i) To a solution of (±)-**19** (130 mg, 0.20 mmol, 1 eq) in DCM (10 mL) at room temperature was added DDQ (888 mg, 3.90 mmol, 20 eq). The mixture was stirred under reflux for 48 h. The mixture was cooled to room temperature, then filtered on a celite pad and concentrated under reduced pressure. The residue was purified by FC (cyclohexane/ EtOAc 7:3) to give 76 mg of (*E*)-5-(2-(2-(4-acetoxyphenyl)-3-(3,5-diacetoxyphenyl)benzofuran-5-yl)vinyl)-1,3-phenylene diacetate as a white sticky solid. Yield: 60% R*_f_*: 0.28 (cyclohexane/ EtOAc 7:3) ^1^H NMR: (300 MHz, chloroform-*d*) δ 7.76–7.71 (m, 2H), 7.62 (d, *J* = 1.3 Hz, 1H), 7.53–7.51 (m, 2H), 7.17–7.09 (m, 7H), 7.07 (d, *J* = 7.7 Hz, 1H), 7.03 (t, *J* = 2.1 Hz, 1H), 6.81 (t, *J* = 2.1 Hz, 1H), 2.31 (s, 15H). ^13^C NMR: (75 MHz, chloroform-*d*) δ 169.3; 169.1; 169.0; 153.9; 151.6; 151.3; 150. 9; 150.8; 139.9; 134.3, 132.3, 130.6, 120.1, 128.2, 127.5, 126.2, 123.9, 121.9, 120.3, 118.1, 116.8, 115.8, 115.0, 114.1, 111.5, 21.2.

ii) To the suspension of (*E*)-5-(2-(2-(4-acetoxyphenyl)-3-(3,5-diacetoxyphenyl)benzofuran-5-yl)vinyl)-1,3-phenylene diacetate (76 mg, 0.115 mmol, 1 eq) in MeOH at 0 °C was added KOH 85% (76 mg, 1.15 mmol, 10 eq). The mixture was stirred at 0 °C for 90 min. and then acidified with HCl 0.1M until pH 1. MeOH was evaporated and residue was diluted with water (5 mL), extracted with EtOAc (3 × 10 mL). The organic phase was washed with water (10 mL) and brine (10 mL), dried over anhydrous Na_2_SO_4_ and concentrated under reduced pressure. The residue was purified by FC (cyclohexane/acetone 1:1) to afford 36 mg of the desired product as a brown amorphous solid. Yield: 70%. R*_f_*: 0.25 (DCM/MeOH 9:1) ^1^H NMR (300 MHz, methanol-*d4*) δ 7.56–7.44 (m, 5H), 7.13 (d, *J* = 16.3 Hz, 1H), 6.93 (d, *J* = 16.3 Hz, 1H), 6.80–6.73 (m, 2H), 6.48 (d, *J* = 2.3 Hz, 2H), 6.43 (d, *J* = 2.3 Hz, 2H), 6.34 (t, *J* = 2.3 Hz, 1H), 6.17 (t, *J* = 2.3 Hz, 1H). ^13^C NMR (75 MHz, methanol-*d4*): δ 158.8, 158.2, 157.8, 153.4, 151.4, 139.7, 134.7, 132.6, 130.6, 128.5, 128.3, 127.3, 122.6, 121.8, 117.2, 115.4, 114.9, 110.5, 107.8, 104.6, 101.6.^13^

**(±)-5,10-bis(4-hydroxyphenyl)-4b,5,9b,10-tetrahydroindeno[2,1-a]indene-1,3,6,8-tetraol (pallidol)** (±)-**12**

To a solution of **1** (1 g, 4.38 mmol, 1 eq) in acetone (21.9 mL) : phosphate buffer pH 8 (21.9 mL) was added a solution of HRP (1.75 mL, 1 mg/mL aqueous solution) at 40 °C and the resulting mixture was stirred for 30 min. Then H_2_O_2_ 30% (0.66 mL) was added to the solution and the mixture was stirred at the same temperature for 1 h. The acetone was evaporated and the aqueous layer was extracted with EtOAc (3 × 50 mL). The combined organic layers were washed with brine, dried over anhydrous Na_2_SO_4_ and concentrated under reduced pressure. The residue was purified by flash column chromatography (DCM/MeOH 85:15) to give 210 mg of the desired product as a brown solid. M.p.: 296 °C dec. Yield: 21%. R*_f_*: 0.15 (DCM/MeOH 9:1). ^1^H NMR (300 MHz, methanol-*d_4_*) δ 6.99–6.87 (m, 4H), 6.68–6.62 (m, 4H), 6.52 (d, *J* = 2.1 Hz, 2H), 6.10 (d, *J* = 2.1 Hz, 2H), 4.46 (s, 2H), 3.72 (s, 2H). ^13^C NMR (75 MHz, acetone-*d_6_*) δ 159.3, 156.3, 155.3, 150.3, 137.7, 129.0, 123.2, 115.8, 103.3, 102.5, 60.5, 53.9.^14^

**(*E*)-4-((±)-3-(3,5-dimethoxyphenyl)-5-(3,5-dimethoxystyryl)-2,3-dihydrobenzofuran-2-yl)phenol** (±)-**8**

To a solution of pterostilbene (100 mg, 0.39 mmol, 1 eq) in acetone (1.9 mL): citrate buffer pH 5 (1.9 mL) a solution of HRP (160 µl, 1 mg/mL aqueous solution) was added at 40 °C and stirred for 30 min. Then H_2_O_2_ 30% (59 µl, 0.573 mmol, 1.47 eq) was added to the solution and the mixture was stirred at the same temperature for 15 min. Acetone was evaporated and the aqueous layer was extracted with EtOAc (3 × 50 mL). The combined organic layers were washed with brine, dried over anhydrous Na_2_SO_4_ and concentrated under reduced pressure. The residue was purified by FC (cyclohexane/EtOAc 7:3) to give 61 mg of the desired product as a white amorphous solid. Yield: 61%. R*_f_*: 0.2 (cyclohexane/EtOAc 3:1). ^1^H NMR: (300 MHz, chloroform-*d*) δ 7.37 (dd, *J* = 8.3, 0.8 Hz, 1H), 7.24–7.19 (m, 3H), 7.02 (d, *J* = 16.2 Hz, 1H), 6.92 (d, *J* = 8.3 Hz, 1H), 6.88–6.78 (m, 3H), 6.62 (d, *J* = 2.2 Hz, 2H), 6.41 (t, *J* = 2.2 Hz, 1H), 6.36 (t, *J* = 2.2 Hz, 1H) 6.34 (d, *J* = 2.2 Hz, 2H), 5.51 (d, *J* = 8.4 Hz, 1H), 4.48 (d, *J* = 8.4 Hz, 1H), 3.81 (s, 6H), 3.75 (s, 6H). ^13^C NMR (125 MHz, chloroform-*d*) δ 161.2, 161.0, 159.8, 156.0, 144.0, 139.8, 132.6, 132.3, 132.1, 130.9, 130.8, 129.1, 128.8, 128.1, 127.6, 126.3, 123.2, 115.6, 109.8, 106.5, 104.3, 99.8, 57.9, 55.5.^15^

**1.3 References**

1. Lee, H. S., Lee, B. W., Kim, M. R., & Jun, J. -G. (2010). Syntheses of resveratrol and its hydroxylated derivatives as radical scavenger and tyrosinase inhibitor. *Bulletin of the Korean Chemical Society*, *31*, 971–975. https://doi.org/10.5012/bkcs.2010.31.04.971

2. Biasutto, L., Marotta, E., Mattarei, A., Beltramello, S., Calicet, P., Salmaso, S., Bernkop-Schnurch, A., Garbisa, S., Zoratti, M., & Paradisi, C. (2004). Absorption and Metabolism of Resveratrol Carboxyesters. *Cellular Physiology and Biochemistry*, *24*, 557–566. https://doi.org/10.1159/000257512

3. Bernini, R., Barontini, M., & Spatafora, C. (2009). New lipophilic piceatannol derivatives exhibiting antioxidant activity prepared by aromatic hydroxylation with 2-iodoxybenzoic acid (IBX). *Molecules*, *14*, 4669–4681. https://doi.org/10.3390/molecules14114669

4. Han, S. Y., Lee H. S., & Choi, D. H. (2009). Efficient total synthesis of piceatannol via (*E*)-selective Wittig-Horner reaction. *Synthetic Communications*, *39*, 1425–1432. https://doi.org/10.1080/00397910802528944

5. El-Deeb, I. Y., Funakoshi, T., Shimomoto, Y., Matsubara, R., & Hayashi, M. (2017). Dehydrogenative Formation of Resorcinol Derivatives Using Pd/C-Ethylene Catalytic System. *J. Org. Chem.*, *82*, 2630−2640 https://doi.org/10.1021/acs.joc.6b03037

6. Polunin, K. E., & Schmalz, -G. H. (2004). Application of Chromium-Arene Complexes in the Organic Synthesis. Efficient Synthesis of Stilbene Phytoalexins. *Russian Journal of Coordination Chemistry*, *30*, 252-261. https://doi.org/10.1023/B:RUCO.0000022800.70211.7d

7. Mattarei, A., Biasutto, L., Romio, M., Zoratti, M., & Paradisi, C. (2015). Synthesis of resveratrol sulfates: turning a nightmare into a dream. *Tetrahedron*, *71*, 3100-3106. http://dx.doi.org/10.1016/j.tet.2014.09.063

8. Pettit, G. R., Grealish, M. P., Jung, M. K., & Hamel, E. (2002). Antineoplastic Agents. 465. Structural Modification of Resveratrol: Sodium Resverastatin Phosphate. *J. Med. Chem.*, *45,* 2534-2542. http://dx.doi.org/10.1021/jm010119y

9. Li, W., He, Shi, X. W., Jia, H., & Zhong, B. (2010). Pan-PPAR Agonists Based on the Resveratrol Scaffold: Biological Evaluation and Docking Studies. *ChemMedChem*, *5*, 1977-1982. https://doi.org/10.1002/cmdc.201000360

10. Lindgren, A. E. G., Öberg, C. T., Hillgren, J. M., Elofsson, M. (2016). Total Synthesis of the Resveratrol Oligomers (±)‐Ampelopsin B and (±)‐ε‐Viniferin. *Eur. J. Org. Chem.*, 426–429 https://doi.org/10.1002/ejoc.201501486

11. Vo, D. D. & Elofsson, M. (2016). Total Synthesis of Viniferifuran, Resveratrol-Piceatannol Hybrid, Anigopreissin A and Analogues – Investigation of Demethylation. *Advanced Synthesis & Catalysis*, *358*, 4085–4092. http://dx.doi.org/10.1002/adsc.201601089

12. Pezet, R., Perret, C., Jean-Denis, J. B., Tabacchi, R., Gindro, K., & Viret, O. (2003). δ-Viniferin, a resveratrol dehydrodimer: one of the major stilbenes synthesized by stressed grapevine leaves. *Journal of Agricultural and Food Chemistry*, *51*, 5488–5492. https://doi.org/10.1021/jf030227o

13. Beneventi, E., Conte, S., Cramarossa, M. R., Riva, S. & Forti, L. (2015). Chemoenzymatic synthesis of new resveratrol-related dimers containing the benzo[b]furan framework and evaluation of their radical scavenger activities. *Tetrahedron*, *71***,** 3052-3058. https://doi.org/10.1016/j.tet.2014.11.012

14. Snyder, S. A., Breazzano, S. P., Ross, A. G., Lin, Y., & Zografos, A. L. (2009). Total Synthesis of Diverse Carbogenic Complexity within the Resveratrol Class from a Common Building Block. *Journal of the American Chemical Society*, *131*, 1753–1765. http://dx.doi.org/10.1021/ja806183r

15. Velu, S. S., Buniyamin, I., & Ching L. K. (2008). Regio- and stereoselective biomimetic synthesis of oligostilbenoid dimers from resveratrol analogues. *Chemistry - A European Journal*, *14*, 11376–11384. http://dx.doi.org/10.1002/chem.200801575
